# Ohmic Heating Extract of Vine Pruning Residue Has Anti-Colorectal Cancer Activity and Increases Sensitivity to the Chemotherapeutic Drug 5-FU

**DOI:** 10.3390/foods9081102

**Published:** 2020-08-12

**Authors:** Meirielly S. Jesus, Ana C. Carvalho, José A. Teixeira, Lucília Domingues, Cristina Pereira-Wilson

**Affiliations:** 1 CEB–Centre of Biological Engineering, University of Minho, 4710-057 Braga, Portugal; merielly_@hotmail.com (M.S.J.); jateixeira@deb.uminho.pt (J.A.T.); luciliad@deb.uminho.pt (L.D.); 2 Department of Biology, University of Minho, 4710-057 Braga, Portugal; anacpcarvalho@gmail.com; 3 CITAB–Centre for the Research and Technology of Agro-Environmental and Biological Sciences, University of Trás-os-Montes and Alto Douro, 5000-801 Vila Real, Portugal

**Keywords:** vine pruning residue, bio-waste valorisation, ohmic polyphenol extraction, colorectal cancer, 5-FU, RKO cells, HCT116 cells, apigenin, quercetin, functional foods

## Abstract

Vine pruning residues are by-products of the wine industry that have not received much attention in the past, in spite of being rich in bioactive compounds. In this study, we aimed to test whether an ohmic extract of vine pruning residue (VPE) has anti-colorectal cancer (CRC) properties, and whether responses differ according with cell’s mutation profile. VPE decreased human CRC cell proliferation, accompanied by DNA effects and cell cycle modulation. VPE also increased cell sensitivity to the chemotherapeutic drug 5-FU. Our results suggest that tumors harboring BRAF mutations may be more responsive to VPE than KRAS mutated tumors. These effects of the extract were not completely reproduced by the most abundant constituents tested individually at the concentrations present in the effective dose of VPE. Globally, our results indicate that VPE, a polyphenol enriched extract produced by ohmic heating of vine pruning residue, has anti-colorectal cancer potential, including sensitizing to a chemotherapeutical drug, and its use in functional foods or nutraceuticals could be exploited in personalized anti colorectal cancer dietary strategies. Valorization of this lignocellulosic residue should encourage bio-waste recycling, adding value to this agricultural by-product and promoting the sustainable use of natural resources.

## 1. Introduction

Environmental concerns have prompted the desire to reduce the impact of the agro-food industry, and transform today’s linear economy into a more efficient circular model. In line with this, the scientific community has been devoting major efforts to the search for bio-waste recycling methodologies that promote sustainable growth, based on the efficient use of natural resources [1,2].

Grapes are a major agricultural product, and according to the 2019 Statistical Report on World Vitiviniculture [3], 77.8 million tons were produced worldwide in 2018, with 57% being used for wine making. The European Union (EU) alone accounts for 45% of the world’s area under vine, corresponding to 3.2 million hectares [4]. Portugal is within the EU’s top five wine producers (considering litres produced per land square kilometre), and has the fourth largest agricultural area under vine after Spain, France, and Italy [3].

Many grapevine by-products, such as grape pomace, seeds, stems, and vine leaves, have been extensively characterized in terms of chemical composition and biological properties [5,6], and have been used as ingredients in dietary supplements [7] and cosmetics [8]. Vine pruning residues, however, remain under-exploited, and are often burned in the fields, contributing to green-house gas emissions, which runs counter to the urgent need for climate change mitigation [9,10]. 

Vine pruning residues constitute valuable biomass that can be used in a biorefinery scheme, considering their high content in antioxidant compounds (phenolic compounds derived from lignin processing) [11,12,13], and as a potential source of xyloligosaccharide prebiotics [14]. They can also be used as biofuels through the hydrolysis and fermentation of cellulose to ethanol [15] or as a source of bioactive polyphenols [16].

Recently, we have reported on ohmic heating, an environmental friendly polyphenol extraction technique, that allowed an increase in phenolic compound extraction from vine pruning residue, relative to extraction at room temperature [16]. In addition to quercetin, which was present in both extracts, hesperidin, ellagic acid and apigenin accounted for the majority of phenolics extracted by ohmic heating (Table 1). 

Resveratrol and quercetin (and several of its derivatives) have been found in many grapevine by-products, together with other compounds [5]. Among the bioactivities attributed to these compounds are antioxidant, antimicrobial and anticancer activity [16,17,18]. Although positive health effects of other wine industry residues have been shown, and pharmaceutical drugs patented and marketed [19], vine pruning extracts have not been explored for this purpose. In the present study, we apply an environmentally friendly method—ohmic heating—for extraction of bioactive compounds from vine pruning residue and explore their colon cancer therapeutic potential.

The anti-colon cancer (anti-CRC) potential of polyphenol enriched ohmic vine pruning extract was tested using two human CRC cell lines, HCT116 and RKO, with different molecular signatures representative of the most common mutation types of CRC, in order to characterize possible molecular targets and mechanisms of action. Moreover, the major individual constituents present in the extract were tested alone, in an attempt to identify possible active principles. To identify possible effects on sensitivity to the chemotherapeutical drug 5-fluorouracil (5-FU), combination treatments were also performed.

## 2. Materials and Methods 

### 2.1. Reagents

Cell culture media (RPMI-1640 and MEM) fetal bovine serum (FBS), antibiotic antimycotic solution, thiazolyl blue tetrazolium bromide (MTT), apigenin (AP), quercetin (Q), ellagic acid (EA), hesperidin (H), etoposide (ET), ribonuclease A (RNase A), propidium iodide (PI), Hoechst 33342, 5-fuorouracil (5-FU), and all reagents not otherwise specified, were purchased from Sigma-Aldrich (St. Louis, MO, USA). SYBR Gold nucleic acid gel stain was acquired from Invitrogen (Paisley, UK). 

### 2.2. Raw Material and Extraction Process of Bioactive Compounds

Vine pruning was harvested in the Portuguese region of Minho (Amares-Braga, Portugal), and samples were dried and crushed as described by Jesus and collaborators (2019) [20]. 

The extraction parameters defined in this study were based on previous results [16,20]. Briefly, the extractions were carried out in a glass cylindrical reactor of 30 cm total length and 2.3 cm of internal diameter, containing two stainless steel electrodes insulated with polytetrafluoroethylene, kept at a constant distance of 7 cm. The ohmic heating extraction of phenolic compounds (vine pruning residue (VPE)) was carried out with a liquid solid ratio of 1:4, 45% hydroalcoholic solution (*v/v*), with electrical conductivity adjusted to 2.3 mS/cm with addition of NaCl (thus guaranteeing a homogeneous current flow), 80 °C, for 60 min under agitation (840.0 V/cm). The room temperature extract (RT) was prepared by placing the vine pruning inside the reactor and maintaining under constant agitation by a magnetic stirrer. At the end of each treatment, the extracts obtained were immediately cooled in an ice bath, and then filtered with a cellulose filter, with a pore size of 0.45 μm. The ethanol was evaporated in an orbital shaker at 40 °C and 150 rpm, and the extracts lyophilized and stored in amber flasks and frozen until use. The phenolic composition of the extracts was determined by Jesus and collaborators (2020) [16], and the major constituents are presented in Table 1.

### 2.3. Cell CuLture and Experimental Conditions 

HCT116 and RKO colon carcinoma cells were kindly provided by Prof. Raquel Seruca (i3S/Ipatimup, University of Porto, Portugal). Cells were maintained at 37 °C in a humidified 5% CO_2_/95% air (*v/v*) atmosphere in RPMI-1640 medium containing 10 mM HEPES, 1 mM sodium pyruvate, 2.0 g/L sodium bicarbonate, 10% (*v/v*) FBS, and 1% (*v/v*) antibiotic antimycotic solution (HCT116 cells) or MEM medium supplemented with 10 mM HEPES, 1 mM sodium pyruvate, 2.2 g/L sodium bicarbonate, 10% (*v/v*) FBS, and 1% (*v/v*) antibiotic antimycotic (RKO cells). Cells were seeded at 1 × 10^5^ cells/mL, 1 day before incubation with extracts/compounds. 

Stock solutions of RT, VPE, AP, Q, EA, H, ET, and 5-FU were made in dimethyl sulfoxide (DMSO), and kept in aliquots at −20 °C. Extracts/compounds were then added to the culture medium just before use, and the final DMSO concentration was never higher than 0.5% (*v/v*); controls received medium with DMSO only.

### 2.4. Cell Viability

In the different experiments, effects on cell viability were estimated by the MTT reduction assay, as previously described [17,21]. Briefly, 2 h before the end of the treatment period, MTT solution was added directly to each well at a final concentration of 0.5 mg/mL. After discarding the medium, the formazan crystals formed were dissolved with a 1:1 (*v/v*) DMSO:ethanol solution, and absorbance was measured at 570 nm with background subtraction at 690 nm. The results were expressed as percentage relative to the control (cells incubated with vehicle). The concentration of extracts that decreased the number of viable cells to 75% (IC_25_) and 50% (IC_50_) relative to control in each cell line were calculated using GraphPad Prism software, version 7.0 (GraphPad Software, San Diego, CA, USA).

### 2.5. DNA Damage

The ability of the VPE and its major constituents to induce DNA strand breaks in HCT116 and RKO cells after 48 h of incubation was evaluated by the alkaline version of the comet assay, as previously described [22,23]. Briefly, cells were collected by trypsinization, embedded in low melting point agarose (0.5% *w*/*v*), and spread on agarose coated slides. Slides were immersed in lysis buffer to expose DNA, and then in electrophoresis buffer to allow alkaline DNA unwinding. In the same solution, electrophoresis was carried out at 4 °C for 20 min at 0.8 V/cm and 300 mA. After neutralization, slides were stained with SYBR Gold nucleic acid gel stain, and analyzed by fluorescence microscopy using the Comet Assay IV analysis software, version 4.3 (Perceptive Instruments Ltd., Suffolk, UK). At least 100 randomly selected nucleoids were scored per sample and the % DNA in comet tail or tail intensity determined. Cells incubated with etoposide (10 μM) were used as positive control for DNA damage.

### 2.6. Cell Cycle

The effects of 48 h incubations with VPE or its major constituents on cell cycle progression of HCT116 and RKO cells was evaluated by flow cytometry, as previously described [24]. Briefly, both floating and attached cells were collected, fixed with cold 70% (*v*/*v*) ethanol, washed with PBS, and incubated for 15 min at 37 °C in the dark, with a staining solution containing 20 μg/mL RNase A and 50 μg/mL PI. Cell cycle was analyzed using a EC800 flow cytometry analyzer (Sony Biotechnology Inc.), counting at least 20,000 single cells per sample, and the percentage of cells in each phase of the cell cycle was calculated using the Watson pragmatic algorithm within FlowJo analysis software (FlowJo, LLC, Ashland, OR, USA). After subtracting the percentage of cells at the sub-G1 phase, the sum of the three phases of cell cycle (G0/G1, S, and G2/M) in each tested condition was equalized to 100%, to compare statistical differences in each phase between samples. Etoposide (10 μM) was used as positive control, as it induces G2/M cell cycle arrest [25].

### 2.7. Cell Death Assessed by the Nuclear Condensation Assay

The effect of VPE, AP, and H, alone or in combination with 5-FU, on HCT116 and RKO cell death (48 h of pre-incubation followed by 48 h of co-incubation), was evaluated by fluorescence microscopy assessing the presence of nuclear condensation, as previously described [26]. Briefly, both floating and attached cells were collected, fixed with 4% (*w*/*v*) paraformaldehyde, washed with PBS, and attached to poly-L-lysine coated slides using a Shandon Cytospin 4 centrifuge (Thermo Scientific, Waltham, MA, USA). Then, the slides were stained with Hoechst 33342, and analyzed by fluorescence microscopy. At least 500 randomly selected cells were counted per sample, and the percentage of apoptotic cells was calculated from the ratio between cells presenting nuclear condensation and the total number of cells.

### 2.8. Statistical Analysis

GraphPad Prism software, version 7.0 (GraphPad Software, San Diego, CA, USA) was used for graphic representation and statistical analysis of the data. Statistical significance was evaluated by one-or two-way ANOVA followed by the Dunnett’s test, as appropriate. Corresponding significance levels were indicated in the figures and respective legends.

## 3. Results

Vine pruning extracts were previously obtained by two extraction methods—room temperature (RT) and ohmic heating (VPE) at 80 °C for 60 min—and their phenolic compound composition characterized [16]. Here, the extracts and their major phenolic constituents were tested, in order to assess their anti-colon cancer activity. The extracts were tested using two cell lines representative of the main genetic profiles of human colon cancer, HCT116 (KRAS mutated) and RKO (BRAF mutated) and IC_50_ and IC_25_ values determined (Table 2; Figure S1). VPE induced higher cytotoxicity than RT, in both cell lines, reflected in the lower IC_50_ and IC_25_ values. RKO cells were more sensitive than HCT116 to both extracts.

The two extracts have been chemically characterized in a previous study [16] and the concentrations of major constituents present in the IC_25_ and IC_50_ doses of VPE were calculated and shown in Table 3. The major phenolic constituents of VPE, quercetin (Q), apigenin (AP), hesperin (H), and ellagic acid (EA) were tested individually in the concentrations at which they are present in the IC_25_ and IC_50_ values of VPE, in an attempt to identify the possible active principle in the VPE extract with regard to anti-colon cancer activity.

**Figure 1:**
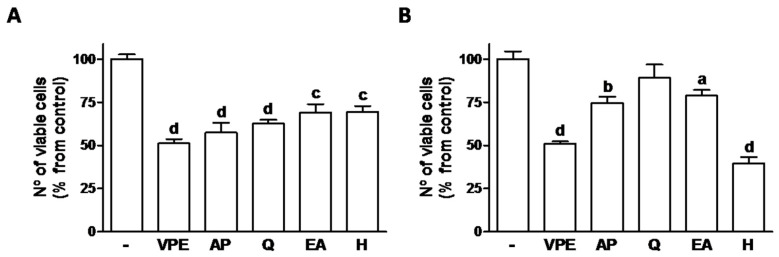
Effect of 48 h exposure to VPE (IC_50_) and of its major constituents (as present in respective IC_50_s–see Table 3) on cell viability of HCT116 (**A**) and RKO (**B**) cells, as measured by the MTT assay. Values are expressed as mean ± SEM of three independent experiments. Letters represent statistical significance: **a** *p* ≤ 0.05, **b** *p* ≤ 0.01, **c** *p* ≤ 0.001, **d** *p* ≤ 0.0001 when compared with control by one-way ANOVA.

The effects on cell viability of both AP and Q were similar to the effects to VPE in HCT116 cell cells whereas in RKO, H was the only compound that alone possessed similar effect to that of the whole extract (Figure 1).

**Figure 2:**
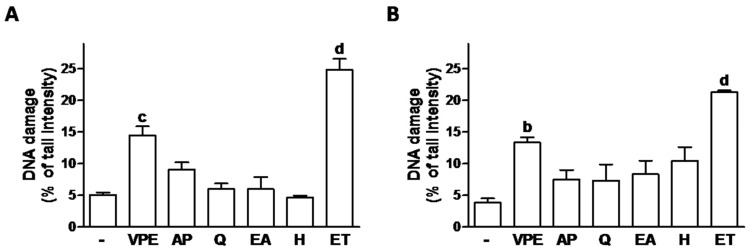
Effect of 48 h exposure to VPE (IC_50_) and of its major constituents (as present in respective IC_50_s–see Table 3) on DNA damage of HCT116 (**A**) and RKO (**B**) cells, as evaluated by the comet assay. ET (10 µ) was used as positive control. Values are expressed as mean ± SEM of three independent experiments. Letters represent statistical significance: **b** *p* ≤ 0.01, **c** *p* ≤ 0.001, **d** *p* ≤ 0.0001 when compared with control by one-way ANOVA.

In order to determine whether the effects on cell viability were due to the induction of DNA damage, the comet assay was used and effects compared to those of etoposide (a known chemotherapeutic agent [27] used here as positive control; Figure 2 and Supplementary Figure S2). VPE showed to have significant DNA damaging effects, although damage was lower than that induced by ET. The individual compounds did not induce significant genotoxic effects (Figure 2).

**Figure 3:**
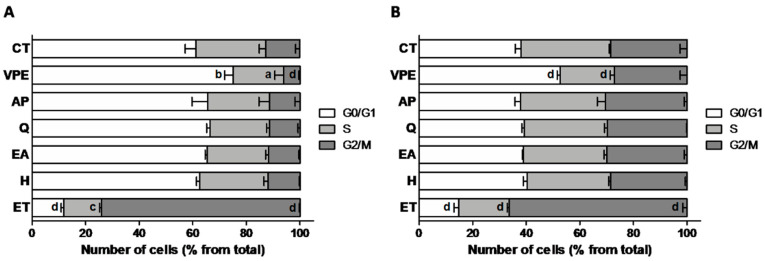
Effect of 48 h exposure to VPE (IC_50_) and of its major constituents (as present in respective IC_50_–see Table 3) on cell cycle of HCT116 (**A**) and RKO (**B**) cells, as assessed by flow cytometry. Values are expressed as mean ± SEM of three independent experiments. Letters represent statistical significance: **a** *p* ≤ 0.05, **b** *p* ≤ 0.01, **c** *p* ≤ 0.001, **d** *p* ≤ 0.0001, when compared with respective phase control by one-way ANOVA.

With regard to cell cycle modulation, the positive control ET produced a significant G2/M arrest in both cell lines, as expected due to its known genotoxic activity [27]. VPE, on the other hand, induced a significant G0/G1 arrest in both cell lines, and the individual compounds did not produce any significant effect relative to control cells (Figure 3 and Supplementary Figure S3). These results are in agreement with the DNA damage detected by the comet assay.

**Figure 4:**
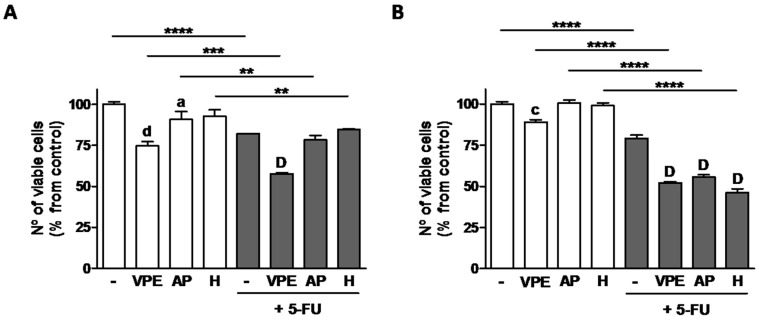
Effect of 48 h exposure to VPE, apigenin (AP), and hesperidin (H), alone and in combination with 5-FU on cell viability of HCT116 (**A**) and RKO (**B**) cells, as measured by the MTT assay. Cells were pre-incubated with the VPE (IC_25_) and corresponding concentrations of AP, or H (see Table 3) for 48, followed by co-incubation with 5-FU (IC_25_) for a further 48 h (**A**&**B**). Cells that were not pre-treated were only exposed to 5-FU for 48 h. Values are expressed as mean ± SEM of three independent experiments. Letters and symbols represent statistical significance: **a** *p* ≤ 0.05, **c** *p* ≤ 0.001, **d** *p* ≤ 0.0001 when compared with control; **D** *p* ≤ 0.0001 when compared with 5-FU; ** *p* ≤ 0.01, *** *p* > 0.001, **** *p* ≤ 0.0001 when compared with respective extract/compound by two-way ANOVA.

When tested in combination with 5-fluorouracil (5-FU), the pharmaceutical drug most frequently used in colon cancer chemotherapy, VPE potentiated 5-FU effects at 48 h co-treatment in HCT116. AP and H, used individually, were less effective. In RKO cells, the combination effects of 5-FU and VPE were more pronounced than in HCT116 at 48 h co-incubation. In addition, both AP and particularly H had similar effects to VPE in reducing cell viability (an indirect measure of proliferation) (Figure 4).

**Figure 5:**
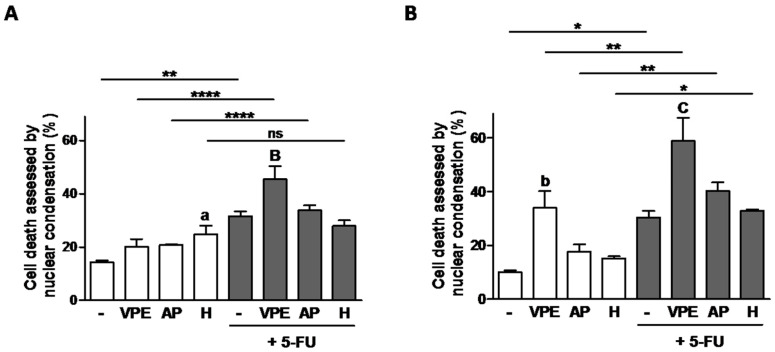
Effect of 48 h exposure to VPE, apigenin (AP), and hesperidin (H), alone and in combination with 5-FU on cell death of HCT116 (**A**) and RKO (**B**) cells, as evaluated by the nuclear condensation assay. Cells were pre-incubated with the VPE (IC_25_) and corresponding concentrations of AP, or H (see Table 3) for 48 h, followed by co-incubation with 5-FU (IC_25_) for further 48 h (**A**&**B**). Cells that were not pre-treated were only exposed to 5-FU for 48 h. Values are expressed as mean ± SEM of three independent experiments. Letters and symbols represent statistical significance: **a** *p* ≤ 0.05, **b** *p* ≤ 0.01 when compared with control; **B** *p* ≤ 0.01, **C** *p* ≤ 0.001 when compared with 5-FU; **ns** *p* > 0.05, * *p* ≤ 0.05, ** *p* ≤ 0.01, **** *p* ≤ 0.0001 when compared with respective extract/compound by two-way ANOVA.

In order to determine if the potentiation of 5-FU by VPE sensitivity was due to the induction of cell death, the nuclear condensation assay was performed and the results are presented in Figure 5. The data shows that relative to 5-FU alone, the combination of 5-FU with VPE increased cell death in both cell lines. In addition, in agreement with the previous results, RKO were more sensitive to the potentiation effect of the combination of 5-FU with VPE than HCT116. Moreover, interesting was the fact that the individual compounds did not seem to induce cell death, although they significantly decreased cell viability, particularly in RKO cells.

## 4. Discussion

Our data show that the ohmic heating extraction method applied to vine pruning residue was particularly effective when compared with extraction at room temperature in extracting phenolic compounds from this winery by-product. Vine pruning residue showed to be an excellent source of apigenin as well as other phenolic compounds. In spite of previous reports on the presence of kaempferol, epicatechin and resveratrol [28,29] in vine pruning residues, extraction of phenolic compound from vine pruning residue has not been sufficiently explored as a means to add value to this by-product of the wine industry. Currently, this bio-waste is mostly disposed of by burning in the fields, which represents a relatively low added value solution with limited return.

In a previous study, we have shown the general anti-cancer potential of vine pruning extracts obtained in different ways, and observed that the normal human colonocyte cell line CCD 841 CoN was significantly less sensitive than cancer cells [16]. With regard to anti-CRC potential, here we show that the extract produced through ohmic extraction of vine pruning residue (VPE) showed better activity than RT extract in both HCT116 and RKO cells. In addition, RKO cells were more sensitive than HCT116 cells, as indicated by the lower IC_25_ and IC_50_ values for RKO. This suggests that colon tumors with the particular genetic profile of RKO (BRAF mutated) may benefit most from the potential applications of VPE in anti-colon cancer strategies than KRAS mutated HCT116-like tumors.

The higher anti-cancer activity of VPE could have been due to the higher concentration of quercetin, a compound repeatedly shown to have anticancer activity (17, 18), or the presence of ellagic acid, hesperidin and apigenin, among other compounds which were absent in RT (Table 1) [16]. Therefore, in an attempt to identify a compound that isolated could be responsible for the effect of the whole extract (active principle), effects of the four major individual compounds were tested in both cell lines at the concentrations present in their respective VPE IC_50_ values (as listed in Table 3), and their effects compared to those of the extract.

In viability assays, AP and Q were the most promising compounds in HCT116 cells, and H in the case of RKO cells, because they seemed to produce similar effects to those of VPE on cell viability. Quercetin, however, in addition to being present in both RT and VPE extracts and just 2 times more concentrated in VPE relative to RT (Table 1), seemed to have somewhat different effects on cell viability in the two cell lines, because, while not having a significant effect in RKO, it significantly decreased cell viability in HCT116. It seems therefore, that Q is not responsible for the effects of VPE.

None of the effects of the individual compounds seemed to be due to DNA damage induction, as demonstrated by the comet assay results. In the comet assay, the percentage of DNA in comet tails (% tail intensity) reflects the presence of DNA beaks. Our data also show the expected DNA damage induction by the positive control etoposide (a known genotoxic agent) (Figure 2 and Supplementary Figure S2) and, to a smaller extent, by VPE.

DNA damage is known to induce cell cycle arrest and/or cell death by apoptosis, and further experiments were performed in order to address effects on these parameters.

Data from cell cycle modulation analyses reflected the observed genotoxicity of ET and VPE. Individual compounds were without effect on cell cycle regulation in both cell lines, which is also in agreement with the absence of DNA damage observed by the comet assay (Figure 3 and Supplementary Figure S3).

In the comparison between VPE and RT, VPE showed, therefore, to be much more efficient than RT against both colon cancer cell lines, possibly due to its much higher phenolic content, although no individual major constituent could be identified as the active principle. This means that the interesting anti colon cancer effect of VPE is due to the complex mixture of compounds present, and not to any its main constituents individually, although some of the individual compounds also showed significant decrease in cell viability.

Due to 5-FU’s significance in the treatment of CRC, combination assays with VPE and two of the most effective individual constituents AP and H with the chemotherapeutic drug were performed, and effects on proliferation and cell death studied in both cell lines. 5-FU alone induced both decreased cell viability and increased cell death in both cell lines, as expected. Interestingly, the combination 5-FU + VPE significantly enhanced 5-FU’s effects on both parameters, which indicates an increased cell responsiveness to the pharmaceutical drug induced by VPE. With regard to the individual compounds, in co-treatment with 5FU, AP and H had similar effects to VPE in RKO cells decreasing viability to a similar extent as VPE. However, those similarities were not present when induction of cell death was considered and effects of individual compounds were also absent in HCT116 cells; VPE showed, therefore, to be more effective in potentiating the response to 5-FU than any of its main flavonoids used individually. This indicates that, although a good source of individual bioactive flavonoids, in particular of AP, VPE as a whole would be more effective in anti-colon cancer dietary strategies, used, for example, in functionalized foods, than its individual compounds.

The combination treatment 5-FU+VPE showed that the effects in RKO were more pronounced than those in HCT116, which is in agreement with the previous responses to VPE alone. Due to the different mutation profiles of the cells used, and the fact that BRAF mutated cells (RKO) responded better to VPE than KRAS mutated cells (HCT116), also in combination treatments with 5-FU, our results suggest that patients with colorectal tumors harboring BRAF mutations would benefit most from combination treatments VPE+5-FU, compared with patients with tumors harboring KRAS mutations. Although this is a significant outcome of our study, future work is required to confirm this trend and clarification of the molecular mechanisms involved. In order to confirm the molecular profile that would benefit the most from VPE and VPE+5-FU, further experiments should test more cell lines with either BRAF or KRAS mutations, and quantify expression levels of phosphorylated ERK (expected to be high in both cell lines, due to the constitutive activation of MAPKinase pathway in both KRAS and BRAF cell lines) and AKT phosphorylation. Since the PI3K/AKT signaling pathway is not expected to be constitutively activated in BRAF mutated cells, but only in KRAS mutated cells, determination of VPE treatment effects on p-AKT levels would also be relevant to establish extract’s effects on the MAPKinase and/or the PI3K/AKT pathways. Furthermore, in spite of the fact that our data suggest an enhancement of the effect of the drug 5-FU by simultaneous treatment with VPE, the establishment of a synergy between VPE and 5-FU will require further experiments.

Taken together, our results show that ohmic heating extraction of vine pruning residue produces a polyphenol enriched extract (VPE) with anti-colorectal cancer potential, including the ability to sensitize cells to 5-FU, a chemotherapeutic drug. The use of VPE in functional foods or nutraceuticals could be exploited in personalized anti colorectal cancer dietary strategies, because higher sensitization to the drug 5-FU is achieved in BRAF mutated tumors, relative to KRAS mutated tumors. Valorization of this lignocellulosic residue of the wine industry should encourage bio-waste recycling, adding value to this agricultural by-product and promoting the sustainable use of natural resources.

## Figures and Tables

**Table 1:** Major phenolic constituents of vine pruning residue extracts obtained by two different extraction methods: at room temperature (RT) and by ohmic heating (vine pruning residue (VPE)).

	RT	VPE
Polyphenols (mg/g extract)		
Ellagic acid	ND	2.23
Apigenin	ND	3.84
Quercetin	1.33	2.87
Hesperidin	ND	1.80
Total Polyphenols *	12	34

For more details see [16]. (*) Includes all compounds identified [16].

**Table 2:** IC_50_ and IC_25_ values of vine pruning residue (VPR) extracts in HCT116 and RKO cells after 48 h of treatment (expressed in µg/mL).

	HCT116		RKO	
	IC_50_	IC_25_	IC_50_	IC_25_
VPR Extracts (µg/mL)				
RT	86.22 ± 3.79	41.17 ± 1.76	36.50 ± 5.47	16.23 ± 1.65
VPE	53.38 ± 1.87 ^d^	24.41 ± 1.31 ^d^	20.91 ± 3.42 ^d^	4.69 ± 1.16 ^c^

Values are expressed as mean ± SD of at least three independent experiments. Letters represent statistical significance: c *p* ≤ 0.001, d *p* ≤ 0.0001 when compared with control (RT) by two-way ANOVA.

**Table 3:** Major constituents as present in the IC_50_ and IC_25_ concentrations of the VPE extract in HCT116 and RKO cells (expressed in µM).

	HCT116		RKO	
	IC_50_	IC_25_	IC_50_	IC_25_
Major Compounds in VPE (µM)				
Apigenin (AP)	4.22	1.93	1.67	0.37
Quercetin (Q)	2.81	1.29	1.09	0.23
Ellagic acid (EA)	2.18	0.99	0.86	0.20
Hesperidin (H)	0.88	0.39	0.34	0.08

## Supplementary Materials

The following are available online at https://www.mdpi.com/2304-8158/9/8/1102/s1, Figure S1: Effect of VPR extracts on cell viability of HCT116 (A&B) and RKO (C&D) cells, as measured by the MTT assay; Figure S2 Representative image obtained by comet assay showing the DNA damage caused by 48h exposure to etoposide (ET, positive control). Figure S3. Representative histograms of the effect of VPE (IC50) and its major constituents on cell cycle of HCT116 (A) and RKO (B) cells for 48 h, as assessed by flow cytometry.
