# Supplementary material for: Ohmic Heating Extract of Vine Pruning Residue Has Anti-Colorectal Cancer Activity and Increases Sensitivity to the Chemotherapeutic Drug 5-FU
Source: Foods. 2020 Aug 12;9(8):1102. doi: 10.3390/foods9081102 (PMC7466249; doi:10.3390/foods9081102)
Supplement: Supplementary file 1 [file foods-09-01102-s001.pdf]

Supplementary Materials

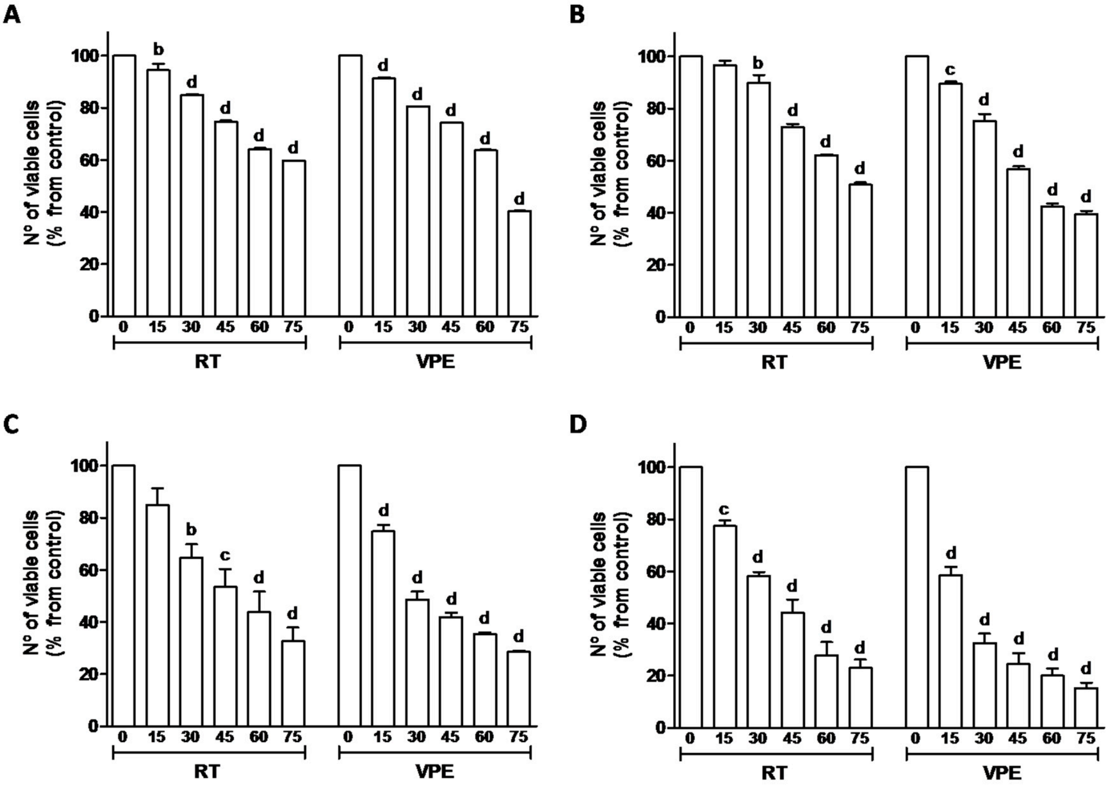

Figure S1. Effect of VPR extracts on cell viability of HCT116 (**A&B**) and RKO (**C&D**) cells, as measured by the MTT assay. Cells were incubated with different concentrations of RT and VPE for 24 h (**A&C**) or 48 h (**B&D**). Values are expressed as mean ± SEM of three independent experiments. Letters represent statistical significance: **b** $p \leq 0.01$, **c** $p \leq 0.001$, **d** $p \leq 0.0001$ when compared with control by one-way ANOVA.

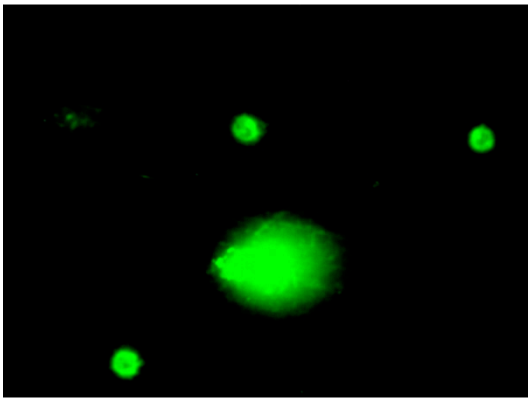

Figure S2. Representative image obtained by comet assay showing the DNA damage caused by 48h exposure to etoposide (ET, positive control).

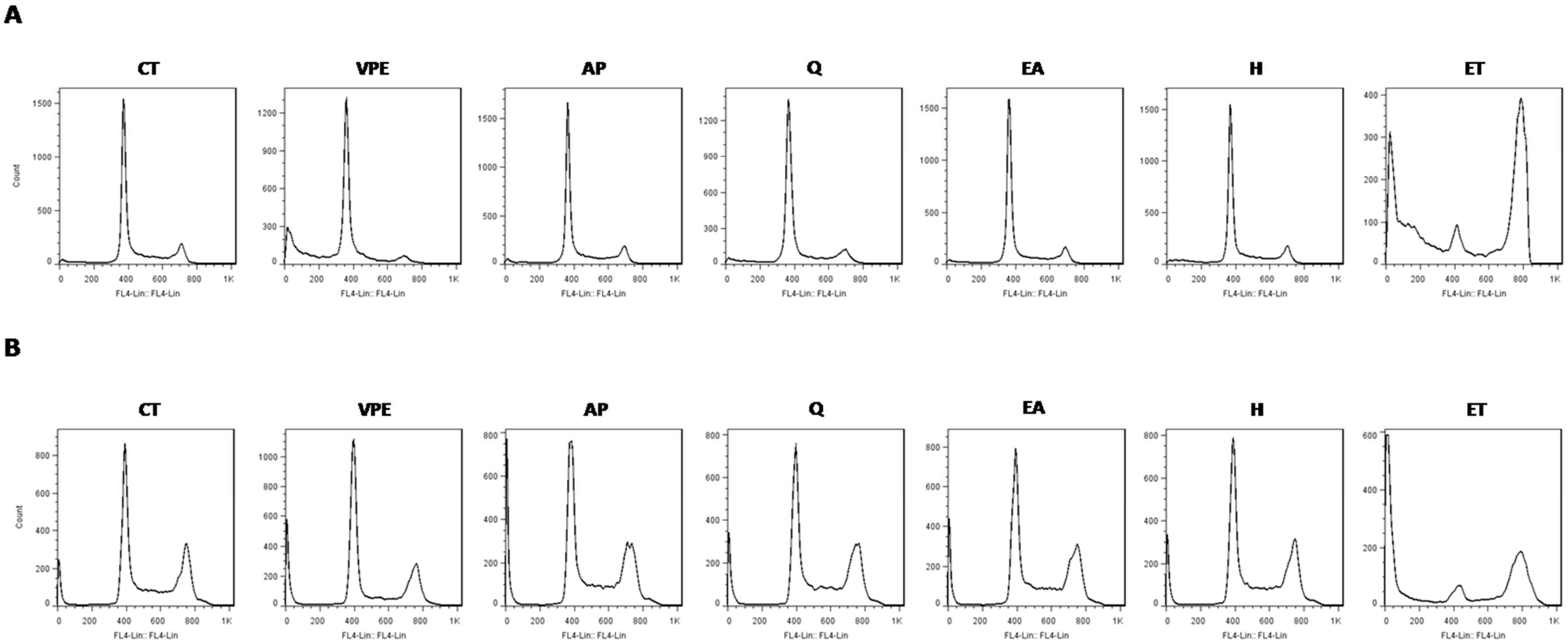

Figure S3. Representative histograms of the effect of VPE (IC_{50}) and its major constituents on cell cycle of HCT116 (**A**) and RKO (**B**) cells for 48 h, as assessed by flow cytometry.
